# The Review on Adverse Effects of Energy Drinks and Their Potential Drug Interactions

**DOI:** 10.3390/nu17152435

**Published:** 2025-07-25

**Authors:** Lukasz Dobrek

**Affiliations:** 1 Department of Pharmaceutical Sciences, Jan Dlugosz University in Czestochowa, 42-200 Czestochowa, Poland; l.dobrek@ujd.edu.pl; 2 Department of Nutrition and Drug Research, Institute of Public Health, Jagiellonian University Medical College, 31-066 Krakow, Poland; lukasz.dobrek@uj.edu.pl

**Keywords:** energy drink, adverse effect, caffeine, cardiovascular abnormalities, drug interactions

## Abstract

**Background:** Energy drinks (EDs) are non-alcoholic, functional beverages sold worldwide in more than 165 countries. These products are very popular and often consumed by children, teenagers, and young adults to improve physical performance, reduce drowsiness, and improve memory and concentration with increased intellectual effort. However, their consumption is associated with an increased risk of various health consequences. **Objectives:** The purpose of this non-systematic review was to discuss the components of EDs and their effects, summarize the AEs reported in the literature associated with the consumption of EDs, and briefly characterize the possible ED-related drug interactions. **Methods**: Scientific evidence was extracted by searching the databases PubMed and Google Scholar. In addition, the reference lists of the retrieved papers were reviewed and cross-referenced to reveal additional relevant scientific evidence. **Results:** The most common ingredients in EDs are caffeine, taurine, glucuronolactone, B vitamins, the vitamin-like compound inositol, and sweeteners (sugar, fructose, glucose–fructose syrup or artificial sweeteners). Although it is difficult to conclusively prove a cause-and-effect relationship between the consumption of EDs and the observed pathophysiological abnormalities, most scientific evidence (mostly clinical case reports) indicates that both occasional and especially chronic use of EDs is associated with the occurrence of numerous adverse effects (AEs). Among these, the best documented AEs are those on the cardiovascular system. It should also be noted that the components of EDs (primarily caffeine) may have drug interactions; therefore, EDs may be an important factor influencing the safety of pharmacotherapy in patients consuming EDs. **Conclusions:** Consuming energy drinks lead to various health problems and may interfere with pharmacotherapy due to the potential development of drug interactions. Due to the widespread availability of EDs, their suggestive advertising aimed at the youngest customers, and ambiguous regulations, new legislative policies are required to limit the widespread consumption of such products and their negative health effects.

## 1. Energy Drink Definition and Estimates of Their Consumption

Energy drinks (EDs) are among the most widely consumed non-alcoholic beverages with a stimulating effect. They fall under the category of functional foods. According to the Functional Food Science in Europe (FUFOSE) definition developed in 1999, a functional food can be said to be “if it has been proven to have a beneficial effect on one or more bodily functions over and above the nutritional effect, which is to improve: health, well-being and/or reduce the risk of disease. These foods must resemble conventional foods in form and show beneficial effects in the amounts expected to be normally consumed from the diet” [1].

Despite the wide use and prevalence of EDs in various countries, there is no clear, common definition of this type of product. According to the European Commission, the term “energy drink” is a commercial term, not legally defined, and refers to products that contain various combinations of substances that are attributed with stimulating effects on the body [2]. In contrast, according to a report by the European Food Safety Authority (EFSA), energy drinks are defined as a variety of soft drinks containing caffeine, taurine, and vitamins (often in combination with other ingredients), marketed for their actual or perceived effects as stimulants, energizers, and performance enhancers [3]. In the country of the author of this manuscript (Poland), according to Article 12l of the Act on Public Health of 11 September 2015: “A beverage with added caffeine or taurine (...) is considered to be a beverage product that is a foodstuff, (...) which contains caffeine in a proportion exceeding 150 mg/L or taurine, excluding naturally occurring substances” [4], whereas the U.S. Centers for Disease Control and Prevention (CDC) defines an energy drink as a product typically containing large amounts of caffeine, added sugars, other additives, and legal stimulants (guarana, taurine, and L-carnitine) that can increase alertness, attention, energy, as well as increase blood pressure, heart rate, and respiration [5].

In 1949, a bottled lemon-lime-flavored, vitamin-enriched soft drink known as Dr. Enuf began to be sold in the US, advertised as relieving feelings of fatigue and exhaustion. The first energy drink (Lipovitan-D) as we understand it today was developed and marketed in Japan in the 1960s. The drink was recommended to abolish fatigue and contained a blend of B vitamins, taurine, and caffeine. The concept of EDs was also developed in other countries, including Europe, where in 1987 one of the most popular “energy drinks” to this day was introduced in Austria as a functional beverage for sports and work—Red Bull. The formula for this product containing taurine, caffeine, and a mix of other ingredients was developed by Austrian businessman Dietrich Mateschitz, who discovered a similar drink during his trip to Thailand. The subsequent years have seen the growing popularity of energy drinks, with nearly 500 new brands of EDs launched worldwide in 2006 (200 new brands were launched in the US alone in the 12 months to July 2007) [6,7].

Energy drinks are widely advertised, especially on television, various online platforms and social media, and their consumption is now portrayed as a “normal” dietary behaviour modification to achieve certain goals requiring increased physical and intellectual exertion [8]. Hence, the ED industry and market dynamics for the sale of such products continue to grow. According to Cognitive Market Research, the global market for energy drinks was estimated at $61,512.2 million in 2024, of which Europe held a market of about 30% of global revenues, with a market size of $18,453.66 million in 2024 [9]. In Poland, the market for annual sales of carbonated beverages reaches PLN 7.8 billion; bottled water, PLN 7.1 billion; juices, beverages, and nectars, PLN 4.9 billion. Energy and isotonic drinks rank fourth in the list of beverage sales, with annual sales of PLN 4.2 billion [10].

According to a systematic review and meta-analysis by Aonso-Diego et al. [11], based on the results of studies conducted in 50 countries involving 1,120,613 volunteers, the estimated global percentage of people who had consumed EDs at least once in the past was 54.7%. In addition, the review found that 43.4% of respondents had consumed EDs in the past 12 months, 32.3% in the past month, 21.6% in the past seven days, and 8.82% consumed EDs daily. An EFSA-commissioned study conducted in 16 EU member states and involving some 32,000 participants found that 68% of adolescents use EDs (with reported percentages of 30% in adults and 18% in children) [3]. Data from the U.S. Department of Health and Human Services, National Center for Complementary and Integrative Health jump to the conclusion that energy drinks and multivitamins are the most popular dietary supplements consumed by U.S. adolescents and young adults. Men between the ages of 18 and 34 consume the most energy drinks, and nearly a third of adolescents between the ages of 12 and 17 drink them regularly [12]. The highest consumption of EDs and the highest sales values of this type of products are observed in the US, Vietnam, Cuba, the UK, Thailand, Mexico, Australia, Germany, Poland, and Saudi Arabia [13]. Thus, Poland is among the 10 countries with the highest consumption of EDs, and the main consumers of such products are young people. A study by Nowak et al. [13] conducted in 2022 with 2629 elementary and high school students showed that 67% of them consume EDs (of which 16.5% consume them frequently—that is, several times a month). A similar study was conducted on a group of 1263 students at various universities in Wroclaw. More than half of the respondents (50.7%) declared at least occasional consumption of EDs, while 17.5% reach for them several times a month and 10.9% several times a week. In addition, 2.4% admitted that they consume EDs daily. The main rationales indicated by respondents who used EDs were that they believed they added energy, reduced drowsiness, had a pleasant taste, improved concentration, combined well with alcohol, and improved memory, while the indicated circumstances for reaching for EDs were sessions and periods of increased study, parties and social gatherings, increased physical activity, vacations and rest time, as well as without any particular circumstance [14]. A study by Zylka et al. [15] conducted in 2018 on a population of high school and college students found that one-third of adolescents (mean age 16.58 ± 1.98) consumed EDs 1–3 times a month, while 5.08% declared consuming EDs daily. The majority of respondents consumed a standard volume of EDs of 250 mL, while 1 in 14 drank EDs of 500 mL or larger. Another paper demonstrating increased consumption of EDs among Polish adolescents was the study of Granda et al. [16], conducted on a group of children and adolescents aged 7–14 participating in after-school sports activities. This study also confirmed the high consumption of EDs among Polish adolescents: 77.7% consumed such drinks 1–3 times a month, 17.3% consumed them between 1 and 4 times a week, and 5.0% of respondents declared consuming EDs from 5 to 6 times a week to even 3 times a day. In summary, the consumption of EDs is high among young adults and especially adolescents, which, in the context of the health consequences described below, creates a major public health problem.

Energy drink overconsumption is associated with the risk of exceeding the intake of the ingredients included in these beverages, as described in detail below in Chapter 2. Considering the most common compound found in ED formulations—caffeine—scientific evidence indicates that the maximum daily intake of this compound for adults should not exceed 400 mg. However, other snacks consumed throughout the day, such as chocolate, can also be sources of caffeine. The amount of caffeine in chocolate varies based on the percentage of cocoa it contains: 100% cocoa chocolate (unsweetened baking chocolate) contains approximately 240 mg of caffeine per 100 g, 55% cocoa (bittersweet) contains 124 mg per 100 g, and 33% cocoa (milk chocolate) contains 45 mg per 100 g. Therefore, it is relatively easy to exceed the recommended caffeine intake by consuming this compound alongside various chocolate snacks and energy drinks. The same concern applies to the risk of overconsumption of other ingredients contained in EDs. Consequently, the risk of developing caffeine-associated side effects from energy drink consumption increases. Importantly, analyses of caffeine intake across different age groups since the 1970s have demonstrated a rising trend in consumption among children and adolescents, who are the primary consumers of energy drinks [17].

The purpose of this non-systematic review was to summarize information on the harmful health effects of EDs, paying particular attention to the most recognized adverse cardiovascular effects in the literature and their pathomechanisms. The author’s intent was also to provide a brief summary on the increased risk of drug interactions that accompany the use of EDs. Scientific evidence was extracted by searching the databases PubMed and Google Scholar. In addition, the reference lists of the retrieved papers were reviewed and cross-referenced to reveal additional relevant scientific evidence.

## 2. Typical Ingredients of Energy Drinks and Their Mechanisms of Action

**Figure 1:**
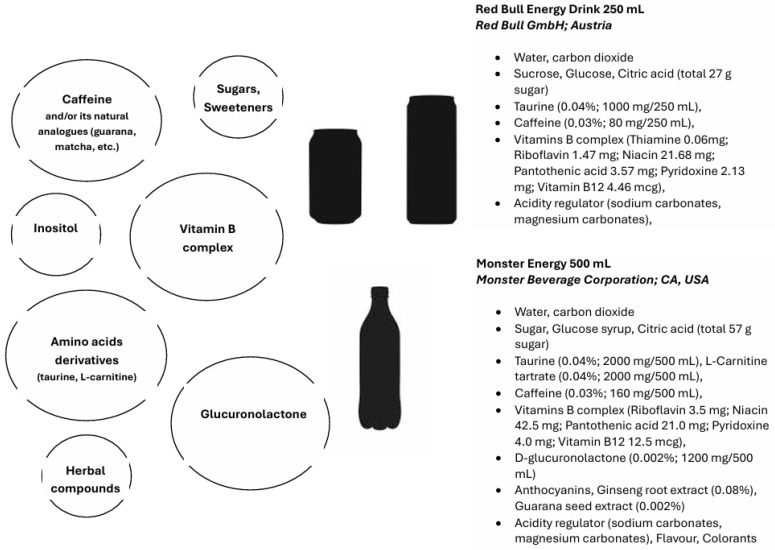
Ingredients of energy drinks.

In general, EDs include ingredients that can be divided into several categories: (1) caffeine and/or its natural analogues (guarana, yerba mate, matcha); (2) vitamin B complex (3) amino acids and their derivatives (taurine, carnitine); (4) sweeteners or their metabolites (sugar, glucuronolactone, synthetic sweeteners); (5) additional herbal compounds (e.g., Ginko biloba, ginseng, green tea) [13]. Consumption of such compounds is expected to improve the body’s performance, increase wakefulness time, and improve memory, concentration, and cognitive abilities. On the other hand, their consumption is associated with significant deleterious effects on physiological functions, especially when combined with alcohol [18,19]. Caffeine, taurine, glucuronolactone, B vitamins, the vitamin-like compound inositol, and sweeteners (sugar, fructose, glucose–fructose syrup, artificial sweeteners) are responsible for most of the effects generated by EDs. These ingredients are found in the greatest quantities in commercially available EDs; the other ingredients are of marginal importance. The composition of a typical commercially available energy drink is shown in Figure 1.

### 2.1. Caffeine

**Figure 2:**
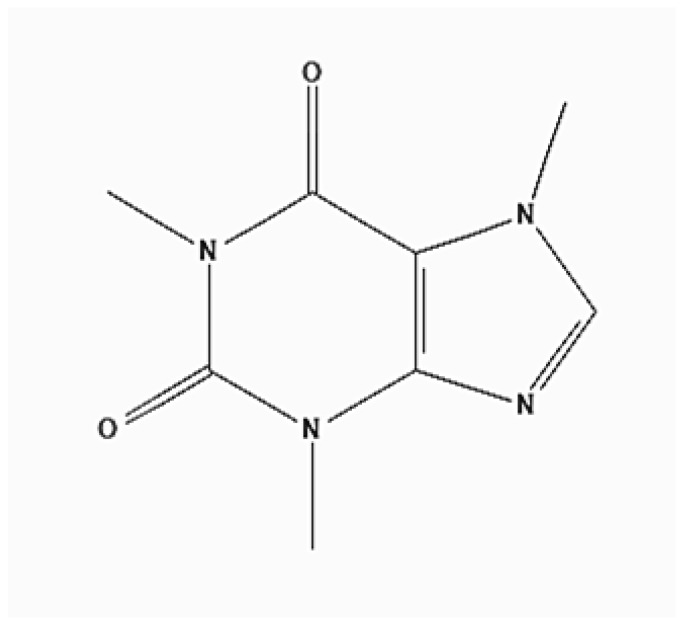
Structural formula of caffeine (author’s own modification; based on source formula published in PubChem database).

Caffeine exhibits stimulant effects at the level of both the central and peripheral nervous systems. The standard volume of a commercially available can of energy drink (250 mL) usually contains 80 mg of caffeine, while the amount of this compound in a 500 mL can or bottle is already 160 mg [20,21]. It should be noted that caffeine is present in numerous other components of a standard diet, so the total daily intake of this alkaloid is higher than just that resulting from the inclusion of energy drink consumption. A standard cup of brewed coffee contains 100 mg of caffeine; a small espresso or a cup of instant coffee—60 mg; and black tea—about 50 mg [20,22,23]. According to a systematic review by Wikoff et al. [24] of the potential adverse effects of caffeine consumption on healthy adults, pregnant women, adolescents, and children, the daily safe dose of caffeine whose combined adult intake of various dietary products was not associated with an increased risk of adverse effects was 400 mg. Evidence also supports that consumption of up to 300 mg of caffeine per day by pregnant women is not associated with adverse reproductive or developmental effects. In children and adolescents, consumption of 2.5 mg caffeine/kg per day is considered safe. According to EFSA’s Panel on Dietetic Products, Nutrition and Allergies, a single caffeine intake of up to 200 mg (about 3 mg/kg for a 70 kg adult) for a total daily intake of 400 mg of caffeine is safe. For pregnant or breastfeeding women, the daily safe dose of caffeine is 200 mg. At the same time, EFSA also proposes a safe level of 3 mg/kg/day as the usual limit of caffeine intake for children and adolescents [25,26]. In terms of a typically available energy drink (containing 32 mg of caffeine/100 mL), this means that a 40 kg person can consume 375 mL of the drink, 562 mL of the drink for a 60 kg person, and 750 mL for an 80 kg person [20]. Thus, the daily consumption of two large 500 mL cans of energy drink or a 1000 mL bottle of such drink by a child/a teenager weighing about 50 kg will result in exceeding the daily caffeine intake limit. Most of the effects, both short- and long-term, induced by EDs, listed in Table 3, are due to the presence of caffeine in such beverages. The structural formula of caffeine is shown in Figure 2 below.

Caffeine induces its effects through a number of mechanisms. One of the main issues of caffeine’s pharmacodynamic description is the blocking of adenosine A1, A2A, and A2B receptors (located in the vascular bed, among others). Caffeine’s antagonistic effect against adenosine receptors prevents adenosine-dependent vasodilatation and increases adenosine concentrations. This leads to increased adrenergic activity, stimulation of alpha- and beta-adrenergic receptors, increased peripheral vascular resistance, and increased activity of the renin–angiotensin–aldosterone system. These mechanisms contribute to the pressor effect of caffeine contained in EDs and contribute to producing positive chrono- (increase in heart rate) and inotropic (increase in myocardial contractility) effects and an increase in blood pressure. The inotropic effect is augmented by caffeine’s inhibition of phosphodiesterase, which increases the concentration of cAMP in the myocardium and enhances the intracellular influx of calcium ions into cardiomyocytes. At very high concentrations (>100 µM), caffeine decreases the sequestration of calcium ions by the sarcoplasmic reticulum and induces vascular smooth muscle contraction (except in the cerebral bed) and tachyarrhythmia [27,28]. Cardiac arrhythmias can be both supraventricular (including atrial fibrillation) and ventricular, conditioned by QT interval prolongation. ED consumption and chronic exposure to an excess of caffeine impairs vascular endothelial function and reactivity and increases the risk of developing prothrombotic incidents and inflammatory reactions in the vessel wall [28]. Excessive adrenergic stimulation induced by caffeine and other stimulant ingredients of EDs also leads to hyperglycemia and hypokalemia, which affects central nervous system function. As a result of the blocking of central A1 adenosine receptors and increased dopaminergic transmission through D2 receptors, ED ingredients increase frontal lobe activity, which reduces fatigue, drowsiness, and improves thought processes. Chronic effects on central A1 receptors lead to the development of the tolerance phenomenon and increased effects on A2A receptors. Caffeine increases neurotransmitter release by abolishing the inhibitory control of acetylcholine in the hippocampus and prefrontal cortex. As a secondary effect, it inhibits thalamocortical glutamatergic neurons by inducing cell activation and stimulating the adenylyl cyclase pathway. Caffeine blocks A2A receptors and reduces the stimulatory effects of adenosine on cAMP. Caffeine can reduce inhibition of striatal dopamine transmission by decreasing the activity of striatal neurons and causing “disinhibition” of thalamocortical projection neurons. Caffeine mimics the effects of dopamine on striatal neurons and causes progressive sensitization of CB1 cannabinoid receptors. In addition, caffeine’s antagonistic effect on A2A receptors results in increased glutamate release, mGlu5 metabotropic receptor activation, and endocannabinoid release. In summary, blocking of adenosine A2A receptors in the striatum has also been linked to the psychoactive properties of caffeine [28]. The combined central effects of caffeine and other psychostimulant components of EDs (e.g., guarana, Yerba mate) can therefore cause “thought chasing”, restlessness, insomnia, tremors, and even anxiety disorders and seizures [28,29].

In the gastrointestinal tract, caffeine has a stimulating effect on salivary secretion and salivary amylase content and contributes to hypergastrinemia, thus increasing gastric juice production. This effect with the disruption of the brain gut axis may contribute to the development of functional dyspepsia. In addition, caffeine relaxes the lower esophageal sphincter, thus predisposing to gastroesophageal reflux disease. This alkaloid also causes the secretion of another enterohormone, cholecystokinin, which stimulates bile production and gallbladder motility and relaxes the sphincter of Oddi, allowing bile and pancreatic juice to drain into the duodenum. Caffeine also enhances colonic motility [30]. Methylxanthines, including caffeine, also exert a diuretic effect, resulting from both an increase in glomerular filtration and a natriuretic effect. By blocking adenosine A1 receptors, caffeine causes dilation of the feeding arterioles in the glomeruli, thereby increasing filtration pressure. In addition, the compound decreases sodium resorption in the proximal nephron and stimulates renin release, resulting in angiotensin-dependent contraction of the draining arterioles [31,32].

### 2.2. Taurine

**Figure 3:**
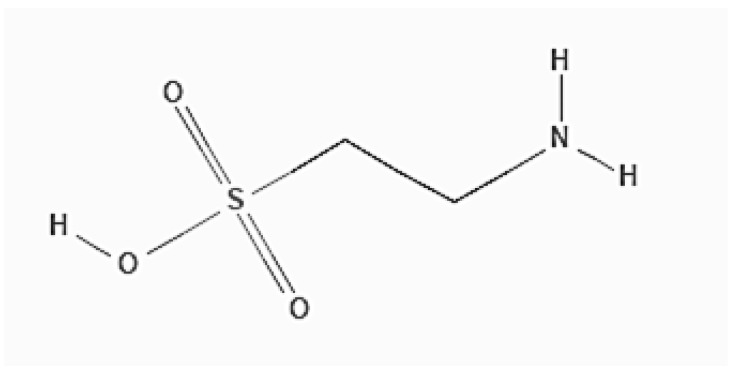
Structural formula of taurine (author’s own modification; based on source formula published in PubChem database).

Another important component of EDs is taurine (2-aminoethanesulfonic acid), a biogenic amino acid synthesized in the course of methionine and cysteine metabolism, containing a sulfone group instead of a carboxyl group. The structural formula of taurine is shown in Figure 3 below. It is an endogenous compound commonly found in the body, especially in plasma and bile, as well as in leukocytes, platelets, heart, brain (especially in the pineal and pituitary glands), and skeletal muscles. Taurine was also found in the liver, kidneys, pancreas, spleen, small intestine, and lungs [33,34]. In addition, taurine is consumed with various foods. A rich source of taurine is shellfish, especially scallops and mussels. Large amounts of taurine can also be found in dark meat from turkey and chicken and other meats, such as veal, lamb, ham, and salami [35]. The average taurine content of EDs is about 4000 mg/L, which, given the most common volumes of EDs consumed, translates into an intake of 1000 mg (250 mL package) or 2000 mg (500 mL package) of taurine [20]. According to EFSA’s assessment, a safe daily dose of taurine is up to 6000 mg/day (equivalent to 100 mg/kg per day for a 60 kg person). This amount did not cause adverse effects [36]. 

Endogenous taurine is a neurotransmitter and neuromodulator with affinity for glycine receptors. Long-term taurine supplementation reduced adrenergic activity and blood pressure values in hypertensive patients [37]. Taurine supplementation also reduced blood pressure values in 24 h ambulatory RR change records [38]. At the same time, taurine, like caffeine, exhibits inotropic-positive properties due to activation of the cardiomyocyte taurine–sodium co-transporter. Intracellular sodium activation activates the sodium–calcium exchanger and leads to an increase in intracellular calcium ion influx, which increases myocardial inotropism [27,39]. At the same time, taurine, in a similar mechanism that promotes calcium influx into skeletal muscles, increases their contractility. It should be noted that the stimulatory effect of taurine on skeletal muscle and the heart disappears with long-term supplementation [21]. Studies also indicate that taurine potentiates caffeine-induced muscle contractility. In summary, the compound exhibits hypotensive activity and enhances muscle contraction via a calcium-dependent mechanism [38]. One of the most important functions of taurine is its anti-inflammatory and oxidative stress-reducing activity (inhibiting, in particular, the oxidation of polyunsaturated fatty acids), whereby the compound exhibits neuroprotective effects and stabilizes the structure of biological membranes [34]. In addition, studies indicate that taurine’s antioxidant properties may be due not so much to the elimination of free radicals, but to the reduction of their generation in mitochondria [39]. Taurine is also conjugated with bile acids synthesized in the liver, forming bile salts that enable the emulsification and digestion of fats. In addition, taurine facilitates detoxification of xenobiotics and drugs in the liver, participating in phase II metabolism and forming conjugates that are excreted into the bile [33,37]. It should be noted that the anti-inflammatory, antioxidant, and cardiovascular protective properties attributed to taurine consumed in the presence of other ingredients contained in EDs are subject to debate and have not been clearly evaluated [23]. In addition, and importantly, the simultaneous consumption of caffeine and taurine included in EDs may have multiple effects on the cardiovascular system, different from the effects expected when these compounds are used separately. A systematic review by Mihaiescu et al. [40] systematized both beneficial and negative effects observed in EDs consumers, conditioned by caffeine and taurine consumption. Among the beneficial effects attributed to the effects of caffeine are improved alertness and cognitive performance, increased physical performance and endurance, and enhanced fat oxidation. These effects are enhanced by taurine, which also increases exercise tolerance and improves intellectual ability, which is also partly due to raising blood glucose and improving the supply of energy substrate to peripheral tissues. These effects are the main reasons why consumers reach for EDs. In contrast, the long-term negative effects resulting from chronic consumption of EDs include increased blood pressure, deleterious effects on the heart function, with the risk of increased arrhythmogenesis, induction of migraines, development of dyspeptic disorders of the upper gastrointestinal tract, diarrhea resulting from accelerated intestinal motility, liver damage, and renal tubular dysfunction, associated with the risk of developing acute kidney injury [40].

### 2.3. D-Glucuronolactone

**Figure 4:**
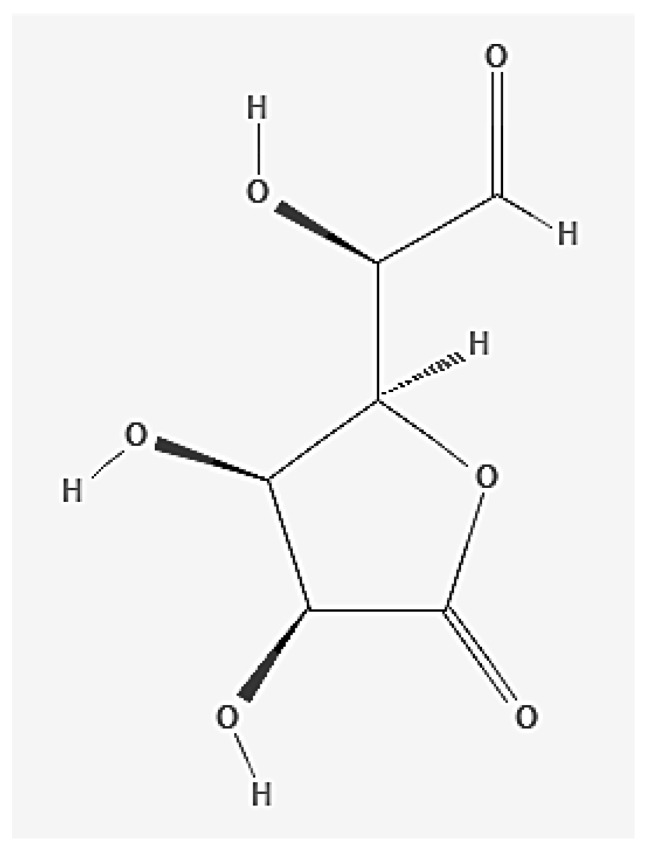
Structural formula of D-glucuronolactone. (Author’s own modification; based on source formula published in PubChem database).

Another ingredient routinely added to EDs is D-glucuronolactone, due to reports that, like caffeine and taurine, it enhances physical and mental performance [21]. Endogenously, D-glucuronolactone is an intermediate metabolite of aldohexoses, converting to, among other things, glucuronic acid—an essential detoxifying compound that is conjugated with xenobiotics (including drugs) in the liver, facilitating their elimination. The structural formula of D-Glucuronolactone is shown in Figure 4 below.

The compound also exerts multifaceted physiological functions: It exhibits antioxidant and anti-inflammatory activity (the compound has been reviewed in clinical settings to assess efficacy in chronic and acute hepatitis and cirrhosis), as well as inhibiting alpha-amylase activity and increasing glycogen stores [41,42]. In addition, an important biological function of glucuronolactone is the participation of this compound in the synthesis of connective tissue. D-glucuronolactone is involved in the synthesis of glycosaminoglycans, glycoproteins, and proteoglycans, which are key components of the extracellular matrix necessary for maintaining the structural integrity and elasticity of cartilage, tendons, and ligaments [21,38]. No significant toxic effects associated with glucuronolactone ingestion have been described to date [38]. The ingestion of a 250 mL energy drink containing an average of 2400 mg/L of D-glucuronolactone provides an average of 600 mg of this compound (this amount doubles if a 500 mL ED is consumed) [20]. The EFSA analysis showed that a daily intake of D-glucuronolactone at a dose of 1000 mg/kg is safe and not associated with significant adverse effects. Thus, the ingestion of an energy drink (250 mL) by a 60-kg person provides about 100 times less D-glucuronolactone with respect to the limit indicated by EFSA [43]. For this reason, the compound should be considered to have no harmful effects, but once again it should be emphasized that the consumption of EDs is associated with the complex delivery of numerous compounds to the body, the presence of which mutually influences the end result, which is difficult to estimate.

### 2.4. B Complex Vitamins

**Figure 5:**
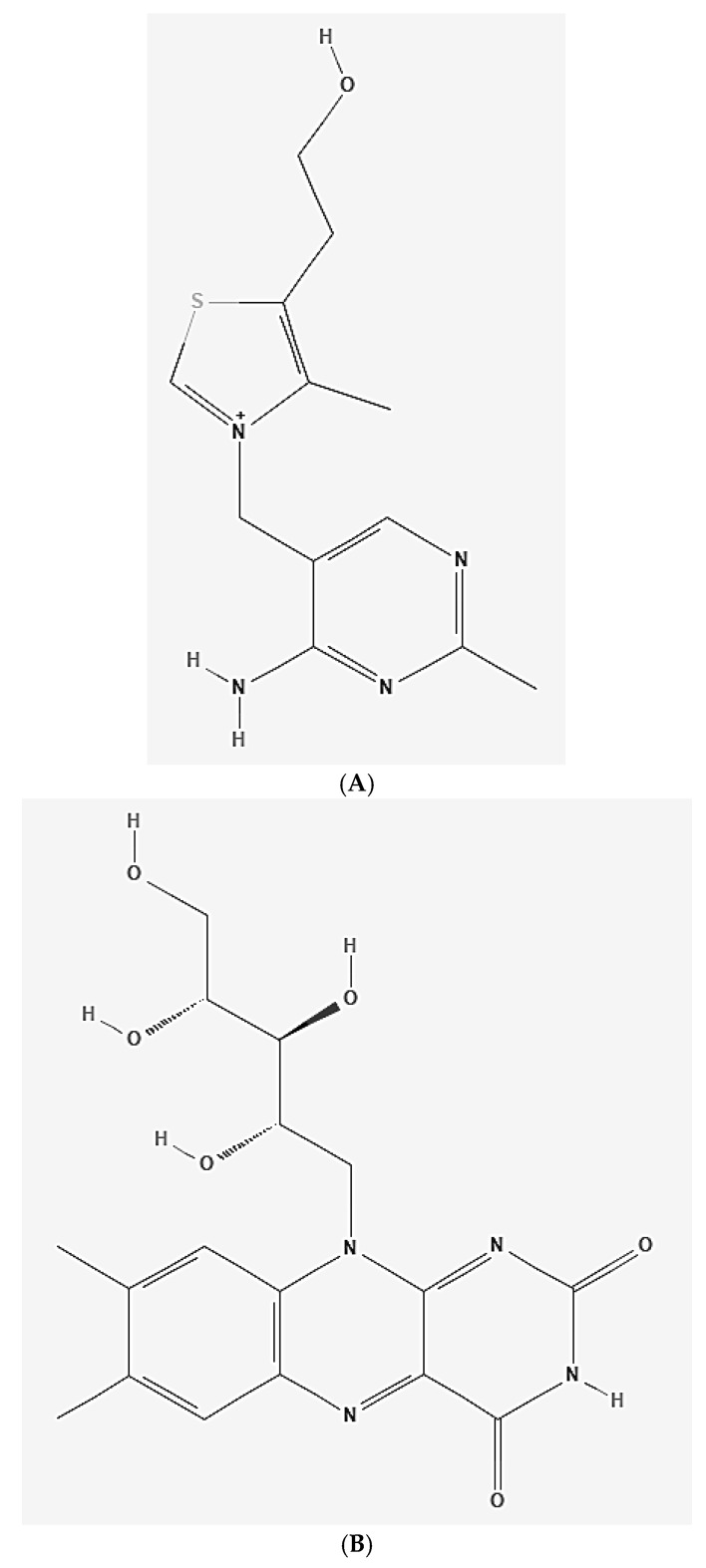

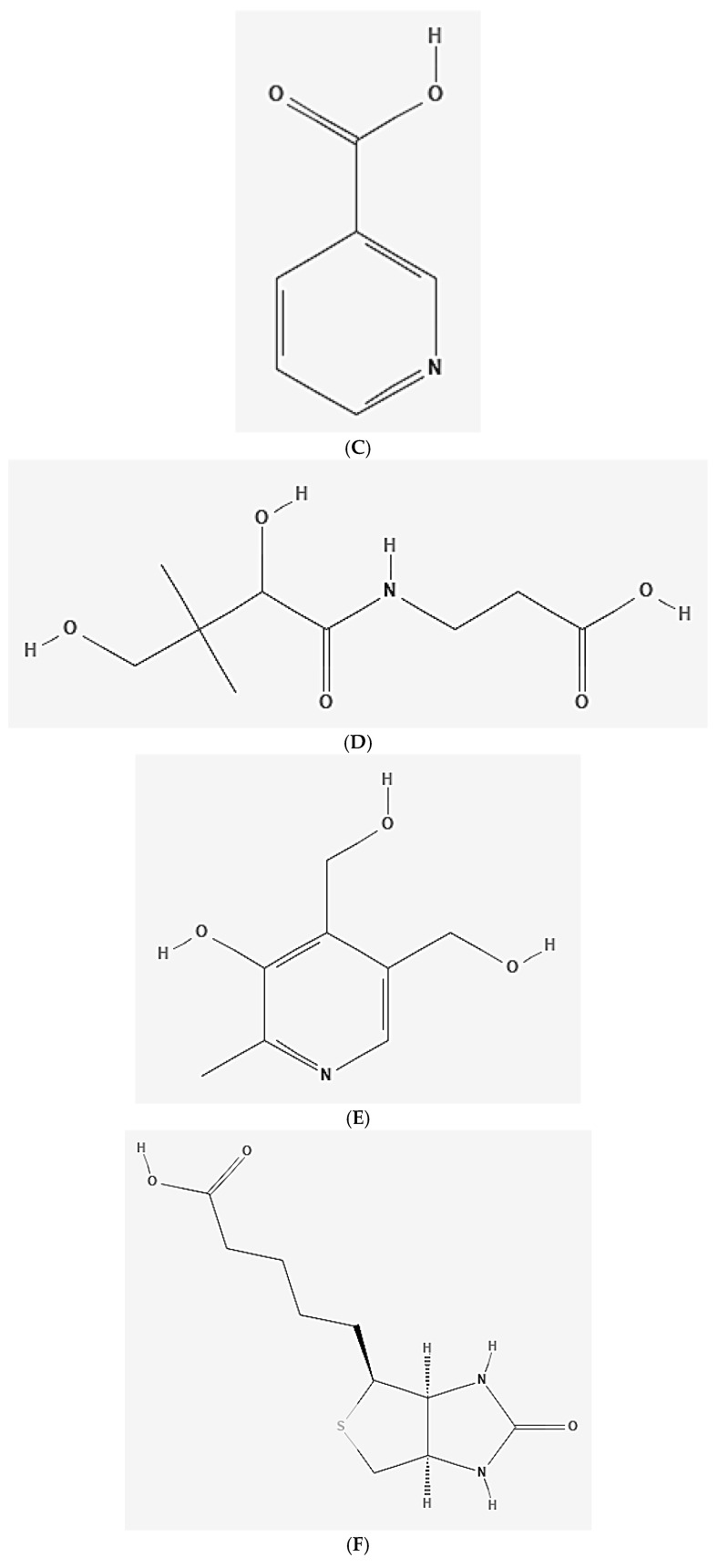

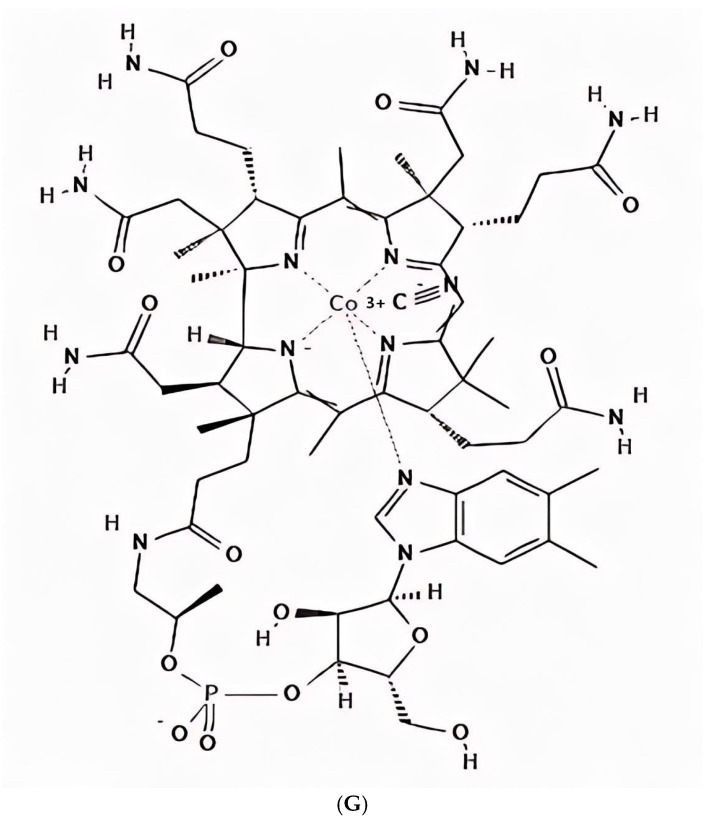
Structural formula of B vitamins. Panel (**A**)—vitamin B1 (thiamin); panel (**B**)—vitamin B2 (riboflavin); panel (**C**)—vitamin B3 (niacin); panel (**D**)—vitamin B5 (panthotenic acid); panel (**E**)—vitamin B6 (pyridoxine); panel (**F**)—vitamin B7 (biotin); panel (**G**)—vitamin B12 (cobalamin) (author’s own modification; based on source formulas published in PubChem database).

Vitamins of the B complex include eight compounds, acting as cofactors for various catabolic and anabolic metabolic reactions occurring especially in cells of the nervous system, associated with the metabolism of amino acids, glucose and fatty acids, participating in the citric acid cycle and the electron transport chain. They also take part in the synthesis of DNA, RNA, axonal transport, and the secretion of many neurotransmitters. They include thiamine (B1), riboflavin (B2), niacin (B3), pantothenic acid (B5), pyridoxine (B6), biotin (B7), and cobalamin (B12). The structural formulas of B vitamins are shown in Figure 5 below. Being water-soluble compounds, they are not stored in the body for long periods of time and must be supplied daily with various dietary products (they are present especially in animal proteins, dairy products, green leafy vegetables, and beans). Vitamin B deficiencies manifest themselves in the form of various neurological disorders and a wide spectrum of pathological conditions (e.g., beriberi disease, pellagra, megaloblastic anemia, peripheral neuropathy, dermatological disorders, and inflammation of the tongue, stomach, and mucous membranes [44].

Recommended daily doses of B vitamins are as follows: for vitamin B2, 1.1–1.3 mg for adults and 1.0–1.3 mg for adolescents; for vitamin B3, 14–16 mg for adults as well as teens; for vitamin B5, 5 mg for adults and teens; for vitamin B6, 1–1.7 mg for adults and 1.2–1.3 mg for teens; and for vitamin B12, 2.4 micrograms for adults and children [45]. According to the EFSA assessment, the daily requirement for thiamine for adults is 0.1 mg/MJ (0.4 mg/1000 kcal). Proportionally, the same reference value, expressed in mg/MJ, is proposed for infants aged 7–11 months, children aged 1 to <18 years, and during pregnancy and lactation, assuming that thiamine requirements versus energy requirements are the same in all population groups [46]. In the case of riboflavin (vitamin B2), the average daily requirement in adults and the associated population reference intake was set at 1.3 and 1.6 mg/day. For children of both sexes aged 1–17 years, the daily requirement ranges from 0.5 to 1.4 mg/day, and the population reference intake from 0.6 to 1.6 mg/day [47]. For vitamin B6, the upper intake level (UL) indicated by EFSA for adults is 12 mg/day (including pregnant and lactating women). The ULs for infants and children are 2.2–2.5 mg/day (4–11 months), 3.2–4.5 mg/day (1–6 years), and 6.1–10.7 mg/day (7–17 years) [48]. For vitamin B12, EFSA indicated an adequate cobalamin intake of 4 µg/day for adults, 1.5 µg/day for infants aged 7–11 months, and 4 µg/day for children aged 15–17 years. During pregnancy and lactation, additional cobalamin intake should be considered (4.5 and 5 µg/day, respectively) due to cobalamin accumulation in fetal tissues and cobalamin transfer into breast milk [49]. In contrast, according to FDA recommendations, the daily requirement for B vitamins in adults is vitamin B3 = 16 mg; vitamin B6 = 1.7 mg; vitamin B12 = 2.4 µg; vitamin B5 = 5 mg [50]. The above data on daily requirements for each vitamin is summarized in Table 1 below.

B vitamins are a common ingredient in EDs and are found in the composition of such products in large quantities (e.g., vitamin B6 (366.9 ± 648 percent of the daily value (%DV)), vitamin B3 (121.44 ± 69.9% DV), vitamin B12 (5244.5 ± 10,474.6% DV), vitamin B5 (113.6 ± 76.6% DV). Thus, it should be concluded that the consumption of EDs is associated with the supply of B vitamins in excess of daily requirements. However, since these are water-soluble vitamins, supplementation via the ED route should not be associated with symptoms of overdose [51].

### 2.5. Inositol

**Figure 6:**
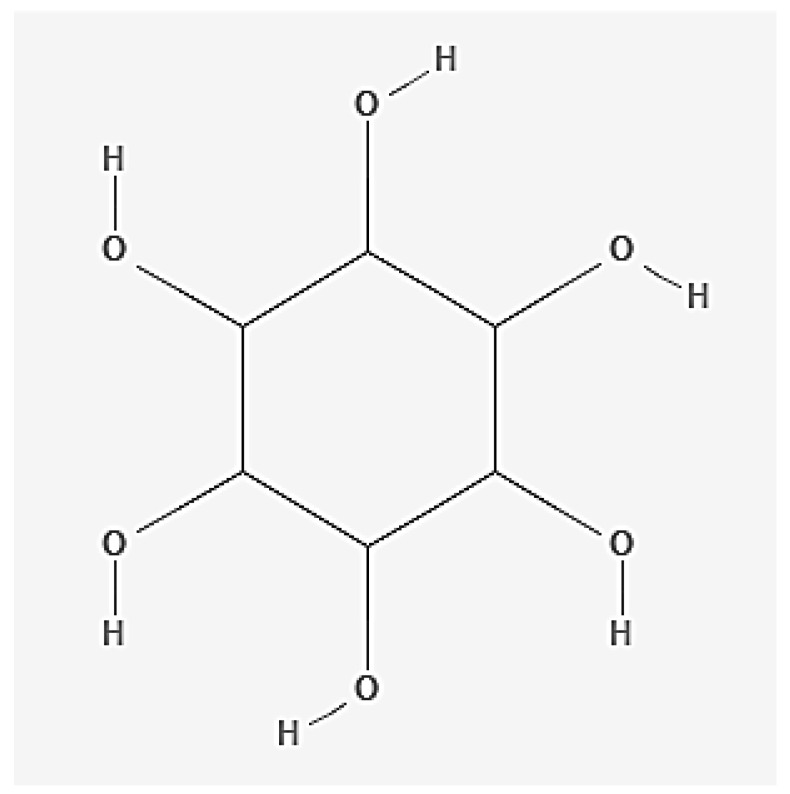
Structural formula of inositol (author’s own modification; based on source formulas published in PubChem database).

Inositol (sometimes called vitamin B8) is a sugar alcohol endogenously found in the form of nine stereoisomers, with myo-inositol being the main form. The structural formula of inositol is shown in Figure 6 below. Inositol, in the form of pyrophosphates, is a compound that improves energy metabolism in cells, including muscle, which is the reason for the use of this compound in EDs. In addition, studies indicate that inositol improves peripheral tissue insulin sensitivity and exhibits anti-inflammatory and antioxidant effects. Abnormalities in inositol phosphate metabolism have been demonstrated in polycystic ovary syndrome and cancer, which has given rise to research into the potential use of this compound in these diseases [38,52,53,54]. In addition, inositol is considered a second messenger and precursor of other second messengers in the central nervous system, studied with inconclusive results in various neuropsychiatric disorders, such as depression, obsessive–compulsive disorder, attention deficit disorder, or Alzheimer’s disease [55]. Inositol is a common dietary ingredient. It is found especially in meat, poultry, fish, and dairy products, as well as citrus fruits, whole-grain flour products, and walnuts, almonds, chia seeds, and flaxseed [56]. The daily allowable intake of inositol through exogenous supplementation has not been clearly defined. Most studies indicate daily inositol supplementation of 2000–4000 mg/day, and this amount given in two divided doses is the proposed upper limit of the supplemented amount accompanying a normal diet [57]. At the same time, studies indicate that the use of inositol at a dose of 12 g/day caused mild gastrointestinal side effects, such as nausea, bloating, and diarrhea. The severity of side effects did not increase with dose [58]. Most energy drinks contain 200 mg of inositol per liter, meaning that 50 mg of inositol is ingested with one 250 mL can. Hence, when considering inositol intake with EDs, based on currently available data, there is no concern about the possibility of selective overdose of this compound.

## 3. Ingredients Not Suitable for Energy Drink Formulation

As a side note, it is important to mention ingredients that should not be included in the formulation of EDs, as their presence may either be incompatible with the primary “energizing,” stimulating effect attributed to the consumption of EDs (e.g., dietary fiber) or prohibited by relevant regulations due to the significant health risks associated with their consumption. Some of them are listed in Table 2 below.

## 4. Adverse Effects Reported in Relation to Energy Drink Consumption

Consumption of EDs is associated with complex effects of the numerous compounds contained in such products, and the final result is more difficult to estimate than would be expected from the analysis of the effects produced by the particular compounds discussed above. However, there is no doubt that both occasional and chronic consumption of EDs is associated with the possibility of some adverse effects (AEs). U.S. data collected by the FDA Center for Food Safety and Applied Nutrition’s Adverse Event Reporting System (CAERS) between 2008 and 2015, summarized by Markon et al. [68], indicate the occurrence of AEs after consuming either a particular energy drink or consuming various products from different manufacturers. When a specific product was consumed, 357 episodes of AEs were reported to CAERS, which occurred in individuals with a mean age of 33.8 years (males—60.2%; females 37.3%). These individuals developed nervous system symptoms (42.6%), psychiatric disorders (17.4%), cardiovascular disorders (23.2%), gastroenterological disorders (29.4%), abnormalities in laboratory results (27.7%), respiratory and mediastinal symptoms (16.5%), and even dermatological manifestations (13.2%). In a significant proportion (41.2%), the reported AEs were of a serious nature, being the cause of hospitalization, and in 14.6% they were even life-threatening. On the other hand, the analysis including the use of EDs of different brands demonstrated 153 reports of AEs from users with a mean age of 39.5 years (men 31.4%; women 66.7%). The most commonly reported AEs in this case were nervous system disorders (37.9%), abnormalities in diagnostic test results (31.4%), gastrointestinal (28.8%), skin and mucosal (23.5%), and cardiovascular (19.0%) symptoms. In the case of chronic ingestion of EDs, the occurred ED-related AEs were the reason for hospitalization in 61.4%, and they were even life-threatening in 5.5%. The work of Markon et al. [68] also discusses AEs observed after EDs, reported to the American Association of Poison Control Centers: National Poison Data System (NPDS) during the same period (2008–2015). The analysis showed that 12,822 AE episodes were likely to be a result of consuming specific EDs. In this case, the disturbances were observed in younger age groups (mean age 13.6 years; males—59.3%; females—40.7%). The most common reported AEs were tachycardia (13.5%), excessive agitation (12.6%), nausea (10.4%) and vomiting (7.5%), dizziness (4.4%), muscle tremor (4.3%), and increased blood pressure (3.0%). The other symptoms: abdominal pain, chest pain, and headaches occurring at a frequency of about 2–3%. In contrast, 931 AEs were reported as resulting from the chronic consumption of various brands of EDs, observed in consumers with a mean age of 22.3 years (males—61.1%; females—38.9%). These AEs also included tachycardia (30.6%), nausea (25.3%), excessive agitation (23.0%), vomiting (17.3%), muscle tremors (11%), chest pain, dizziness (8.6%), and increased blood pressure (7% each). Headache and abdominal pain were reported in about 3–4% [68].

The systematic review by Nadeem et al. [69] covering 32 studies (13 studies with a control group, including 7 randomized trials and 6 cross-sectional studies, and 19 non-comparative studies) published between 2007 and 2018 (a total of 96,549 participants) also summarizes possible AEs associated with ED consumption. The mean age of the study participants was 15.2 years (range 11–63); men accounted for 52.1%. The vast majority of study participants (76.7%) reported consuming EDs < 1 ED/week, 20.6% consumed > 1 ED/week, and 1.2% drank at least 1 ED daily. The characteristics of the AEs revealed in the review are shown in Table 3 below.

However, it should be emphasized that the Nadeem et al. review [69] also included studies without a control group, so it is difficult to unequivocally consider a causal relationship between all the described AEs and ED consumption as certain. In addition, it should also be noted that many of the reported AEs (e.g., restlessness, nervousness, excitement, insomnia, muscle tremors, chaotic thought and speech psychomotor agitation, cardiac arrhythmias) can be considered to be caused by the effects of excessively consumed caffeine, also including sources other than EDs. A systematic review by Ali et al. [70] also aimed at the description of the adverse health effects caused by EDs. Based on a final selection of 43 clinical cases published between 1980 and 2014, the authors found cardiovascular and neurological disorders as the most common AEs. In about one-third of the analyzed cases, various arrhythmias were observed. The other disorders included coronary vasospasm, aortic aneurysm dissection (three cases each), QT interval prolongation, cardiac arrest, development of symptoms of acute cardiomyopathy (two cases each), development of STEMI, coronary artery thrombosis, tachycardia associated with orthostatic reaction, and increased hypertension (one case each). Among the nervous system disorders, seizures (six cases), excessive psychomotor agitation (three cases), and suicide attempt (one case) were demonstrated. Considering the possible pathomechanisms of the observed AEs, the authors once again postulated that they were mainly related to caffeine action. However, they also pointed out that this explanation is not unarguable due to the fact that EDs are often consumed along with alcohol or used in circumstances inherently associated with increased adrenergic stimulation (e.g., playing sports, excessive work-related physical exertion, or during social gatherings) and these factors may also contribute to AE development [70]. On the other hand, it is clear that children and adolescents are the most vulnerable populations to develop adverse health effects associated with the consumption of EDs.

## 5. Harmful Effects of Energy Drinks on the Cardiovascular System

**Figure 7:**
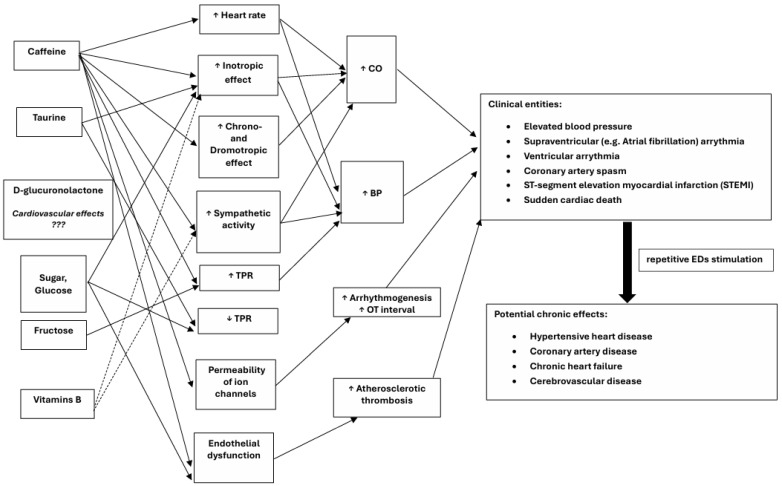
Acute and chronic cardiovascular disorders associated with energy drink consumption, including the postulated pathomechanisms [71,72,73]. Legend: dashed arrow—potential effects due to beneficial effects of B vitamins on nerve fiber function and cardiac energy metabolism; TPR—total peripheral resistance; CO—cardiac output; BP—blood pressure.

In summary, although it is difficult to unambiguously assess the cause-and-effect relationship between ED use and observed adverse health effects without question, the most documented AEs associated with EDs are those affecting the cardiovascular system. Detailed studies evaluating the effects of EDs on the cardiovascular system provide inconclusive and often contradictory results. Some of them are collectively discussed in review type papers, for example, Higgins et al. [71], Grasser et al. [72], Somers et al. [73] or Voskoboinik et al. [74]. However, most studies indicate that consumption of EDs leads to episodes of increased blood pressure and accelerated and increased cardiac contractility. Statistically, in healthy individuals, about 1–2 h after ED ingestion, there is an increase in systolic blood pressure by 6–10 mmHg, diastolic blood pressure by 3–6 mmHg, heart rate by about 3–7 beats/minute, and QT interval prolongation up to 22–25 ms. In addition, the use of EDs facilitates the occurrence of episodes of atrial fibrillation or ventricular tachycardia (especially in people with Brugada syndrome). The abuse of EDs by healthy individuals (consumption of 2–8 cans per day) has been casuistically associated with episodes of coronary artery spasm, development of thrombotic lesions in the coronary arteries, and STEMI-type myocardial infarction [71]. As a result of long-term consumption of EDs and the chronicization of the pathomechanisms outlined below, increased arrhythmogenesis, development of ischemic heart disease, and ultimately chronic circulatory failure are expected to develop. Cardiovascular disorders associated with the consumption of EDs, along with the proposed pathomechanisms, are shown in Figure 7 below.

## 6. Energy Drink Consumption and the Risk of Drug Interactions

EDs ingredients can enter into numerous drug interactions, both pharmacokinetic and pharmacodynamic. The compound with the best documented risk of causing interactions is caffeine. This compound can affect the pharmacokinetic profiles of other orally administered drugs at various stages, that is: absorption, distribution, metabolism, and elimination. Caffeine may change the absorption of numerous weak acid drugs through complexation reactions, via dipole–dipole forces or hydrogen bonding between the polarized carbonyl groups of caffeine and the hydrogen atom of acid drugs. Ultimately, this can lead to a decrease in the bioavailability of selected drugs (such a relationship has been described, for example, for the oxalate salt form of escitalopram and numerous common neuroleptics). The literature also indicates the decreased absorption of iron and L-thyroxine in the presence of caffeine [75]. Caffeine also increases gastric secretion by affecting taste type 2 bitter receptors (TAS2Rs), which are located in the mouth and parietal cells lining the body and floor of the stomach [76]. An excessive production of caffeine-dependent gastric hydrochloric acid can affect the degree of absorption of concomitantly used drugs by degrading the drug, converting the drug substance into an insoluble form, and altering its dissolution rate. In the case of drugs that are weak acids, acidic gastric contents reduce their degree of ionization, and this phenomenon may increase their absorption by diffusion. Such a relationship has been described for acetylsalicylic acid, paracetamol, ketoprofen, and levodopa [75]. Moreover, caffeine’s effects on the gastrointestinal tract are broader—the compound increases bile and pancreatic juice secretion in a cholecystokinin-dependent mechanism and enhances colonic motility, which may accelerate the biliary excretion of drugs [30]. At the distribution stage, caffeine’s effect on increasing the tightness of the blood–brain barrier is important as it may impede the passage of important drugs into the central nervous system, including memantine and donepezil used in the pharmacotherapy of Alzheimer’s disease. Caffeine inhibits peripheral decarboxylase activity, thereby increasing the blood concentration of levodopa and its central conversion to dopamine. The best studied pharmacokinetic process currently affected by caffeine is the influence on drug metabolism. Caffeine is 95% metabolized by the hepatic cytochrome CYP1A2, which, additionally, is characterized by polymorphisms that affect the clearance rate of other compounds metabolized with this enzyme. The homozygous individuals for the CYP1A2 *1A/*1A genotype are fast caffeine metabolizers, while homozygous carriers of the e*1F allele (or the C polymorphism) are slow caffeine metabolizers, resulting in longer caffeine exposure [77]. Caffeine, being a CYP1A2 substrate, competes with other compounds for this isoenzyme. Hence, its use with some drugs that are also metabolized with CYP1A2 is associated with the risk of increasing their blood concentrations and prolonging their duration of action [75,78]. These drugs are listed in Table 4 below.

Caffeine and other methylxanthines also affect glomerular filtration, thereby influencing the elimination of drugs and their metabolites with urine. Caffeine antagonizes A1 receptors of afferent arterioles, which contributes to their vasodilatation, increase in glomerular blood flow, and, consequently, filtration pressure. In addition, blocking A1 and A2A receptors of proximal tubules reduces sodium resorption. These mechanisms lead to an increase in diuresis [79]. Thus, high doses of caffeine are regarded to increase renal clearance and promote more rapid excretion of many drugs and their metabolites from the body.

Caffeine contained in EDs also enters into pharmacodynamic interactions of both synergism and antagonism. A synergistic reaction, expressed as an increase in psychostimulant and cardiovascular stimulant effects, is observed with the combined use of caffeine and sympathomimetics (adrenergic drugs), amphetamine-derived agents, and cocaine. In addition, caffeine enhances the effects of convulsant compounds. Thus, taking large doses of caffeine in conjunction with ED abuse may be associated with a risk of convulsant effects, mostly in patients who take bupropion or tricyclic antidepressants. In addition, caffeine can reduce blood clotting. Consumption of caffeine together with the use of antiplatelet drugs (e.g., clopidogrel), anticoagulants (e.g., warfarin), or non-steroidal anti-inflammatory drugs (NSAIDs; e.g., diclofenac, ibuprofen, naproxen) reducing coagulation may increase the risk of bruising and bleeding. Caffeine, due to its impairment of glucose tolerance and decreased insulin sensitivity, also exhibits antagonistic effects towards hypoglycemic drugs, which is obviously aggravated by the significant sugar load in EDs. Caffeine also antagonizes the effects of sedative and sleep medications; for example, benzodiazepines and “Z” drugs [78,80].

In the case of other ingredients of EDs, their potential to generate drug interactions is far less well documented compared to caffeine. Taurine may also be a component of a pharmacokinetic interaction at the metabolic stage due to its inhibitory effect on the cytochrome CYP2E1. This cytochrome metabolizes some endogenous compounds (e.g., polyunsaturated fatty acids, steroid hormones), small-molecule xenobiotics (e.g., ethanol, carbon tetrachloride), and a relatively small number of drugs for which it is mostly a complementary pathway of metabolism. These include isoniazid, paracetamol, cis-platinum, amide topical anesthetics (lidocaine, prilocaine), halogenated inhaled anesthetics (“flurans”), fluoxetine, theophylline, and some anticonvulsants (phenobarbital, felbamate) [37,81,82]. Some EDs also contain additional ingredients, such as ginseng or ginkgo biloba. Ginsenosides exhibit a characteristic pharmacodynamic interaction, enhancing the effects of antiplatelet and anticoagulant drugs; thus, the use of ginseng contributes to prolonging bleeding and clotting times. Ginseng phytochemicals also produce a hypoglycemic effect; therefore, the use of preparations containing ginseng potentiates the effects of hypoglycemic drugs [37,83]. Similarly, components of Ginkgo biloba also potentiate the effects of antiplatelet and anticoagulant drugs and increase the risk of bleeding, mostly when they are combined with NSAIDs. The attenuation of the anticonvulsant effect of valproic acid and increased risk of serotonin syndrome after antidepressants from the selective serotonin reuptake inhibitor and monoamine oxidase inhibitor groups have also been described in situations when these drugs were combined with Ginkgo biloba preparations [84,85,86]. In summary, the consumption of EDs is associated with a risk of drug interactions due to the documented potential of the most relevant components of EDs (caffeine, to a lesser extent taurine, ginseng, and Ginkgo) to enter into various interactions, both pharmacokinetic and pharmacodynamic. This fact rationalizes the avoidance of consuming EDs by patients receiving chronic treatment, especially NSAIDs, anticoagulants, hypoglycemics, sedatives, hypnotics, sympathomimetics, antidepressants, and anticonvulsants. At the same time, it should be emphasized that there is a lack of studies evaluating the comprehensive impact of EDs on the safety of pharmacotherapy and the risk of the occurrence of clinically significant drug interactions.

## 7. Conclusions: The Need for Appropriate Legislation to Reduce the Widespread Availability of Energy Drinks

Data in the literature indicate that both incidental and especially chronic consumption of EDs is associated with various adverse effects. Therefore, taking into account the harmful effects of EDs on health and the fact that these products are readily consumed by children, teenagers, and young adults, it is necessary to strive for the widespread introduction of policies restricting unrestricted access to EDs. At the moment, there are no uniform legal solutions developed to regulate the marketing of these products, especially in the context of free access to these products.

In Poland (the country of the author of this review), the current Law on Public Health of 11 September 2015, (article 12l) defines a caffeinated energy drink as a product in the form of a beverage that contains caffeine in an amount more than 150 mg/L (with the addition of taurine), excluding naturally occurring substances. At the same time, according to Article 12m of the aforementioned law, the sale of such beverages to persons under 18 years old, distributing such beverages in schools providing educational activities for children, and the possibility of buying such beverages in self-service vending machines are prohibited [4]. Similar restrictions related to the age of consumers also exist in other countries. In 2024, Lithuania was the first EU country which banned the sale of EDs containing at least 150 mg/L of caffeine to consumers aged <18 years old. Similar policies are in force in Latvia, Hungary, and the United Arab Emirates. In Sweden and Norway, EDs are available in pharmacies, allowing for some oversight of their use, while Denmark and Turkey have banned the marketing and sale of EDs altogether [87,88]. A detailed summary of policies on the issue of sales and marketing of EDs and tax burdens designed to discourage consumers from frequent purchases of such products in various countries can be found in the review by Rostami et al. [89]. In a number of countries with as yet unregulated legalization of EDs (e.g., the U.K., Serbia, and Czechia), there is an ongoing debate about limiting the availability of these products. Similarly, in the U.S., physicians affiliated with the American Medical Association are calling for the recognition of EDs as harmful products comparable to alcohol and cigarettes, banning their sale to children and adolescents (however, there is currently no legislation in the U.S. that meets such demands). There are also legislative initiatives in various countries to restrict advertising content related to EDs aimed at children and adolescents [87,88,89,90]. It should also be mentioned that in the European Union there are product-specific labeling rules applicable to energy drinks, enshrined in Regulation (EU) No. 1169/2011 on the provision of food information to consumers [91]. According to this regulation, the labeling of energy drinks should include the information “High caffeine content. Not recommended for consumption by children, pregnant and breastfeeding women”, followed by the amount of caffeine per 100 mL in parentheses. These regulations constitute harmonized European law and are directly applicable in all EU member states, including Poland [91]. In addition, in Poland, some health warnings are placed on the packaging of EDs, such as: “Consume responsibly”, “Consume in moderation. Do not mix with alcohol”, or “A balanced diet and healthy lifestyle is recommended”. Similarly, the U.S. FDA has not developed specific regulations for EDs, considering these products as “generally safe”, and requires them to be labeled accordingly. ED labels must list all ingredients, including caffeine content, and provide information on serving size, calories, and amounts of key nutrients. Moreover, their labels must contain health warnings against excessive consumption, especially for individuals sensitive to caffeine, pregnant women, and those with certain medical conditions [92]. The introduced regulatory initiatives reflect a growing awareness of the potential risks associated with ED consumption and the need to protect public health. Given the many concerns about the harmful effects of EDs on health, some authoritative [71] or institutional (e.g., the American College of Sports Medicine [29]) recommendations for the rational use of such products are also elaborated. They are mentioned in Table 5 below.

In summary, the widespread consumption of energy drinks, especially by children and young adults, in the context of their harmful effects on health, is a growing public health problem that requires global legalization and standardization. The adverse effects of EDs manifest primarily as numerous cardiovascular and nervous system disturbances and can occur even after incidental consumption. The pathomechanisms of AEs occurring after EDs are a result of the individual ingredients of EDs, especially caffeine, taurine, B vitamins, and glucuronolactone. It should also be emphasized that these ingredients may have drug interactions, which reduces the safety of pharmacotherapy. Comprehensive studies evaluating the effects of especially chronic consumption of EDs on numerous physiological functions are still lacking. Most of the evidence present in the literature comes from case-based clinical observations. This rationalizes the undertaking of further studies, including cohort studies, to expand current knowledge regarding the pathophysiology and consequences of energy drink consumption.

Regulatory authorities should implement and promote policies aimed at reducing the overconsumption of energy drinks, particularly among children and young adults. In addition to the existing restrictions in many countries, such as bans on the sale of EDs to individuals under the age of 18, comprehensive educational campaigns should be launched. These campaigns should focus on promoting healthy lifestyles and highlighting the harmful health consequences related to ED consumption, specifically targeting children, teenagers, and young adults (who are the primary consumers of these products), but also healthcare professionals. Furthermore, similar to regulations on other stimulants like tobacco and alcohol, consideration should be given to imposing additional taxes on EDs to discourage their purchase. Additionally, the composition and permissible ingredient content of EDs, especially caffeine, should be clearly defined. Finally, the targeting of suggestive advertising for EDs towards children and adolescents should be restricted.

## Figures and Tables

**Table 1:** Daily requirements for B vitamins, according to various recommendations.

Vitamin	Munteanu, C.; Schwartz, B. B [45]	EFSA [46,47,48,49]	FDA [50,51]
B1		All: 0.4 mg/1000 kcal	
B2	Adults: 1.1–1.3 mg Teenagers: 1.0–1.3 mg	Adults: 1.3–1.6 mg Teenagers: 0.5–1.6 mg	
B3	Adults and teenagers: 14–16 mg		Adults: 16 mg
B5	Adults and teenagers: 5 mg		Adults: 5 mg
B6	Adults: 1.0–1.7 mg Teenagers: 1.2–1.3 mg	Adults: 12 mg *	Adults: 1.6 mg
		4–11 months: 2.2–2.5 mg/day *	
		1–6 years: 3.2–4.5 mg/day *	
		7–17 years: 6.1–10.7 mg/day *	
B12	Adults and teenagers: 2.4 mcg	Adults: 4 mcg	
		7–11 months: 1.5 mcg	Adults: 2.4 mcg
		Children > 15 years: 4 mcg	

NOTE: *—expressed as upper daily intake level.

**Table 2:** Commonly unsuitable or prohibited ingredients of energy drinks [59,60,61,62,63,64,65,66,67].

Ingredient	General Intended Use	Status	Remarks
Dietary fiber Inulin	Gastrointestinal health Satiety	Ingredient inconsistent with the claimed and expected effect of EDs	- Fiber is not a source of quick energy and does not contribute to the stimulatory effect typically expected from energy drinks - Most forms of fiber (especially insoluble) impair the clarity, texture, and shelf stability of liquid products
Ephedrine	Potent stimulant	Prohibited (FDA, EFSA)	Consumption is related to increased cardiovascular risk
Cannabinoids THC/CBD	Feelings of well being Euphoria Quiet and reflective mood	Not authorized in most countries for recreational use	Subject to drug regulations and medical use
Kratom (*Mitragyna speciosa*)	Mood stabilizer Thymoleptic	Prohibited in U.S. and EU	Consumption is related to increased cardiovascular risk, seizures, severe liver toxicity
Yohimbine	Fat burner Stimulant	Prohibited or restricted (FDA, EU-EFSA)	The compound is characterized by a narrow therapeutic index; consumption associated with increased risk of developing hypertension, anxiety, seizures
Bitter Orange (*Citrus aurantium*, synephrine)	Weight loss Stimulant	Permitted with restriction in U.S.; caution in EU (EFSA)	Concerns similar to that found for ephedrine
Kava Kava (*Piper methysticum*)	Anxiety Relaxation	Prohibited/not authorized in EU (EFSA), Canada; not “Generally Recognized As Safe” in EDs by U.S. FDA	Serious hepatotoxicity concerns
Gotu Kola (*Centella asiatica*)	Cognitive supporter Neuroprotection	Moderate use in supplements is generally considered safe, but no specific authorized health claims exist (EFSA, FDA) Use in EDs is generally not authorized due to lack of safety data for these products	Potential liver toxicity; lacks safety for beverages
Senna (*Cassia angustifolia*)	Laxative	The use of Senna in dietary supplements is not authorized in the EU and it is not prohibited in dietary supplements in the U.S.	- Causes diarrhea and dehydration; inappropriate for stimulant beverages - Senna is authorized as a medicinal product (an over-the-counter (OTC) laxative in the EU and U.S.

**Table 3:** Adverse effects reported by energy drink drinkers [69].

System	Adverse Event	Rate in Total Population [%]	Rate in Pediatric Population [%]
			Rate in Adult Population [%]
Nervous system	Headache	18.4	20.9
			13.6
	Dizziness	12.8	10.0
			35.0
	Tremors	11.4	8.1
			20.6
	Slurred speech	32.0	N/A
			32.0
	Walking problems	29.7	N/A
			29.7
	Disturbed coordination	36.9	N/A
			36.9
	Visual disturbances	12.3	14.6
			11.5
	Seizures	1.1	1.0
			1.1
	Depressive mood	23.0	23.1
			13.1
	Agitation/anxiety	23.1	25.6
			18.7
	Irritability	24.0	28.1
			9.6
	Suicidal ideation/attempts	19.8	19.8
			N/A
	Insomnia/sleep-related problems	34.5	35.4
			24.7
Cardiovascular	Tachycardia	26.2	12.5
			56.6
	Palpitations	20.0	17.5
			20.7
	Chest pain	10.3	19.6
			4.9
	Arrhythmia	4.3	1.4
			9.7
	Dyspnea	13.8	17.1
			11.3
Gastrointestinal	Abdominal pain	14.6	14.5
			7.6
	Stomach upset	18.7	9.3
			21.6
	Low appetite	17.3	17.3
			N/A
	Increased salivation	14.0	N/A
			14.0
Urinary	Kidney pain	0.8	N/A
			0.8
	Increased urination	12.9	16.4
			13.6
Musculoskeletal	Muscle tension/pain/twitching	14.0	14.4
			10.3
Other events	Restlessness/shaking hands	25.1	19.3
			29.8
	Jolt and crush	22.6	6.1
			32.9
	Rapid speech	34.5	34.6
			34.4
	Dehydration	18.6	20.8
			16.1
	Fatigue	12.5	0.6
			21.8
	Weakness	28.9	28.9
			N/A
	Heat intolerance	14.9	N/A
			15.9

N/A—not available.

**Table 4:** Drugs associated with the increased risk of reduced metabolism after energy drink consumption due to the possible caffeine-related interactions [75].

Class of Drug	Generic Names of the Drugs
Antidepressants	Fluvoxamine Amitriptyline Clomipramine Mianserin Imipramine
Antipsychotics	Clozapine Olanzapine Haloperidol
Cardiovascular drugs and anticoagulants	Lidocaine Mexiletine Propafenone Verapamil Warfarin
Cholinesterase inhibitors	Tacrine
Hypnotics	Melatonin Zolpidem
Local anesthetics	Ropivacaine
Bronchodilators	Theophylline
Quinolones	Enoxacin

**Table 5:** Recommendations related to the consumption of energy drinks [29,71].

Limitations related to age and specific physiological or pathophysiological conditions	EDs should not be consumed by: Children, adolescents aged <18 years old Pregnant women ≥18 years old Breastfeeding women Individuals with hypersensitivity to caffeine Individuals ≥18 years old with incriminating cardiovascular history: elevated blood pressure, increased heart rate, increased QT interval, cardiac arrhythmia, coronary artery stenosis Individuals ≥18 years old taking medications chronically Patients with diabetes
Restrictions related to the manufacturing, place of purchase and distribution	EDs should not be available in schools and other institutions providing educational and care services for children Manufacturers should not promote excessive consumption of EDs, as there is a close relationship between their excessive consumption and ED-related adverse effects
Advertising restrictions	EDs should not be advertised to children or any other vulnerable populations (listed above)
The need to educate society about the harmful effects of consuming energy drinks	Children, teenagers, and young adults should be widely educated, especially in schools and universities, about the harmful effects of EDs on health, especially when consumed regularly Health care providers and athletic or personal trainers should educate their patients or clients about the rational use of EDs and their potential adverse effects
Additional recommendations	EDs should be avoided before, during, and after strenuous activities EDs should not be consumed close to bedtime (up to 6 h before bedtime) to avoid insomnia EDs should not be mixed with alcohol EDs contain high levels of carbohydrates and calories and may contribute to obesity development; thus, they should be monitored by individuals who are trying to lose or maintain body weigh
